# Folliculogenesis and steroidogenesis alterations after chronic exposure to a human-relevant mixture of environmental toxicants spare the ovarian reserve in the rabbit model

**DOI:** 10.1186/s13048-024-01457-6

**Published:** 2024-06-28

**Authors:** Sara El Fouikar, Nathalie Van Acker, Virginie Héliès, François-Xavier Frenois, Frank Giton, Véronique Gayrard, Yannick Dauwe, Laila Mselli-Lakhal, Delphine Rousseau-Ralliard, Natalie Fournier, Roger Léandri, Nicolas Gatimel

**Affiliations:** 1 ToxAlim (Research Center in Food Toxicology), Université de Toulouse, INRAE, ENVT, INP-Purpan, UPS, Toulouse, France; 2 Plateforme Imag’IN, Service d’anatomopathologie, CHU Toulouse, Institut Universitaire du Cancer-Oncopole de Toulouse, Toulouse, France; 3 GenPhySE (Génétique Physiologie et Système d’Elevage), INRAE, Université de Toulouse, INPT, ENVT, Castanet-Tolosan, France; 4 Pôle Biologie-Pathologie Henri Mondor, AP-HP, Inserm IMRB U955, Créteil, France; 5 Université Paris-Saclay, UVSQ, INRAE, Jouy-en-Josas, 78350 BREED France; 6 Ecole Nationale Vétérinaire d’Alfort, BREED, Maisons-Alfort, 94700 France; 7 Athérosclérose et macrophages: impact des phospholipides et des fonctions mitochondriales sur l’efflux du cholestérol, Lip(Sys) Université Paris Saclay, UFR de Pharmacie, Orsay, EA 7357, 91400 France; 8 Laboratoire de Biochimie, AP-HP (Assistance Publique-Hôpitaux de Paris), Hôpital Européen Georges Pompidou, Paris, 75015 France; 9 Médecine de la Reproduction, Hôpital Paule de Viguier, Centre Hospitalier Universitaire de Toulouse, Toulouse, France; 10 DEFE (Développement Embryonnaire, Fertilité et Environnement) UMR1203 Inserm, Universités Toulouse et Montpellier, CHU Toulouse, Toulouse, France

**Keywords:** Combined exposure, Folliculogenesis, Ovarian function, Endocrine disruptors

## Abstract

**Background:**

Industrial progress has led to the omnipresence of chemicals in the environment of the general population, including reproductive-aged and pregnant women. The reproductive function of females is a well-known target of endocrine-disrupting chemicals. This function holds biological processes that are decisive for the fertility of women themselves and for the health of future generations. However, insufficient research has evaluated the risk of combined mixtures on this function. This study aimed to assess the direct impacts of a realistic exposure to eight combined environmental toxicants on the critical process of folliculogenesis.

**Methods:**

Female rabbits were exposed daily and orally to either a mixture of eight environmental toxicants (F group) or the solvent mixture (NE group, control) from 2 to 19 weeks of age. The doses were computed from previous toxicokinetic data to reproduce steady-state serum concentrations in rabbits in the range of those encountered in pregnant women. Ovarian function was evaluated through macroscopic and histological analysis of the ovaries, serum hormonal assays and analysis of the expression of steroidogenic enzymes. Cellular dynamics in the ovary were further investigated with Ki67 staining and TUNEL assays.

**Results:**

F rabbits grew similarly as NE rabbits but exhibited higher total and high-density lipoprotein (HDL) cholesterol levels in adulthood. They also presented a significantly elevated serum testosterone concentrations, while estradiol, progesterone, AMH and DHEA levels remained unaffected. The measurement of gonadotropins, androstenedione, pregnenolone and estrone levels yielded values below the limit of quantification. Among the 7 steroidogenic enzymes tested, an isolated higher expression of *Cyp19a1* was measured in F rabbits ovaries. Those ovaries presented a significantly greater density/number of antral and atretic follicles and larger antral follicles without any changes in cellular proliferation or DNA fragmentation. No difference was found regarding the count of other follicle stages notably the primordial stage, the corpora lutea or AMH serum levels.

**Conclusion:**

Folliculogenesis and steroidogenesis seem to be subtly altered by exposure to a human-like mixture of environmental toxicants. The antral follicle growth appears promoted by the mixture of chemicals both in their number and size, potentially explaining the increase in atretic antral follicles. Reassuringly, the ovarian reserve estimated through primordial follicles number/density and AMH is spared from any alteration. The consequences of these changes on fertility and progeny health have yet to be investigated.

**Supplementary Information:**

The online version contains supplementary material available at 10.1186/s13048-024-01457-6.

## Background

Humans are inevitably exposed to a plethora of environmental chemicals throughout their life. Among these, approximately one thousand are suspected to possess endocrine disrupting properties [1]. Notably, female reproductive function is a significant target of endocrine disruptors [2] because of the determinant role the ovary [3] and the hormonal signals play in its regulation through the hypothalamus-pituitary-ovarian (HPO) axis and possibly also through the hepato-ovarian axis [4]. Laboratory and clinical studies have provided abundant evidence that these chemicals have deleterious impacts on the ovaries and more generally on the female reproductive organs, including the uterus and the vagina [2]. However, obstacles remain in reprotoxicity assessment due to the complexity of women’s exposomes. The latter is characterized by combined [5] and evolutive [6–8] exposure to xenobiotics. However, most existing studies have investigated the effects of individual compounds. Although these studies are informative and contribute to deciphering mechanistic pathways, their direct applicability to real-world scenarios of human exposure to a combination of environmental chemicals may be limited. In fact, the additivity of chemical effects cannot be assumed because interactions can occur within a mixture. Few in vivo reproductive toxicity studies have investigated mixtures of chemicals, most of which have focused on chemicals from the same families, such as phthalates [9–11], brominated flame retardants [12–14], pesticides [15, 16] or fertilizers [17, 18]. They confirm the threat of mixtures exposure for the ovary on different levels (see exhaustive review [19]) but additional studies need to be performed on mixtures combining compounds from different chemical families. Currently, the literature dealing specifically with the female reproductive effects of combined mixtures is limited [20–30] and lacks relevance in terms of tested doses, thus limiting its usefulness for extrapolating to human health.

The period of folliculogenesis was targeted in this study because it constitutes a crucial biological process for female reproductive capability as follicles serve as the ovary’s functional unit and fulfil essential roles through their development. The initial pool of primordial follicles represents the ovarian reserve (OR) which shapes the length of reproductive life. As they develop, follicles then contribute to ovarian steroidogenesis and therefore hormone regulation within the HPO axis. Moreover, folliculogenesis is tightly linked to oogenesis [31] culminating in the release of a fully mature fertilizable oocyte upon completion of follicular development [32]. Since folliculogenesis spans from before birth to menopause [33] it can be challenged by women life events and environmental fluctuations. Given the major implications of folliculogenesis mentioned above, the impacts of exposure to xenobiotics need to be assessed to protect women’s reproductive capacity. Especially because ovarian function is regulated by genetic, paracrine, and endocrine factors and the latter are directly threatened by xenobiotics with endocrine disrupting properties.

The present study aimed to evaluate the consequences of an oral exposure during folliculogenesis to a mixture of eight environmental contaminants. This study was part of a research project named FEDEXPO (Folliculogenesis and Embryo Development EXPOsure to a mixture of toxicants) whose entire protocol has been previously presented [34]. The evaluation was carried out using a rabbit model because it is a relevant model for toxicological studies and has interesting characteristics regarding folliculogenesis and preimplantation embryo development [35]. Notably, in this specie, the assembly of primordial follicles ends approximately two weeks after birth, allowing us to study the effects of a direct in vivo exposure during the entire folliculogenesis process [36]. Previous toxicokinetic data enabled our team to compute the oral doses to be administered to rabbits to reproduce the serum concentrations of environmental chemicals in the range of those encountered in pregnant women [37]. This experimental strategy constitutes the novelty of the study since, to our knowledge, it is the first to investigate such relevant doses of a highly diverse mixture of toxicants during folliculogenesis. To decipher the impact of exposure during folliculogenesis, on adult ovarian functions, we studied after ovulation induction, parameters assessing both folliculogenesis and steroidogenesis. Classical ovarian histology will allow to study the dynamic between the different follicular stages. Immunohistochemistry will complete these data by further investigating cellular proliferation and DNA fragmentation within ovarian regions of interest. Biochemical dosages will evaluate the ovarian hormones profile while gene expression analysis of steroidogenic enzymes will help identifying potential endocrine disruptions. Finally, a macroscopic ovarian analysis post ovulation will give answers regarding ovulating abilities after exposure to the mixture.

## Materials and methods

### Chemicals

All the materials for the preparation of the solutions were made of glass or polypropylene. 2,2’,4,4’-Tetrabromodiphenyl ether (BDE-47) and hexachlorobenzene (α-HCB) were purchased from Toronto Research Chemicals (North York, Ontario, Canada). Perfluorooctanesulfonic acid (PFOS), di-(2-ethylhexyl) phthalate (DEHP), perfluorooctanoic acid (PFOA), p,p′-dichlorodiphenyldichloroethylene (DDE), β-hexachlorocyclohexane (β-HCH), and bisphenol S (BPS) potassium salt were purchased from Sigma‒Aldrich (St. Louis, MO, USA).

### Animals

The animal experiments were authorized by the French Ministry of Research under the number APAFIS No. 14787-201804201607003 v3 and responded to the local ethical committee approval, including the rules of the directive 2010/63/EU, notably ensuring the 3R rules. Thirty-two nulliparous New Zealand White rabbit females (INRA 1777 line) from the Experimental Unit PECTOUL (Pompertuzat, France) were included in this study. The animals were housed and bred to avoid unintentional contamination with potential endocrine-disrupting chemicals, as described in the FEDEXPO study protocol [34]. The rabbits were fed ad libitum commercial feed (Stabifibre, Terrya, Rignac, France) and water, the latter being supplied by glass bottles with a stainless-steel pipette. The light cycle was 12HL:12HD, and the room temperature was maintained at approximately 18 °C. The females included in this study were 2 weeks old. From weaning to 10 weeks of age, the females were bred in the same cages by group of 3. After 10 weeks of age, they were dispatched one per cage.

### Experimental design

**Fig. 1:**
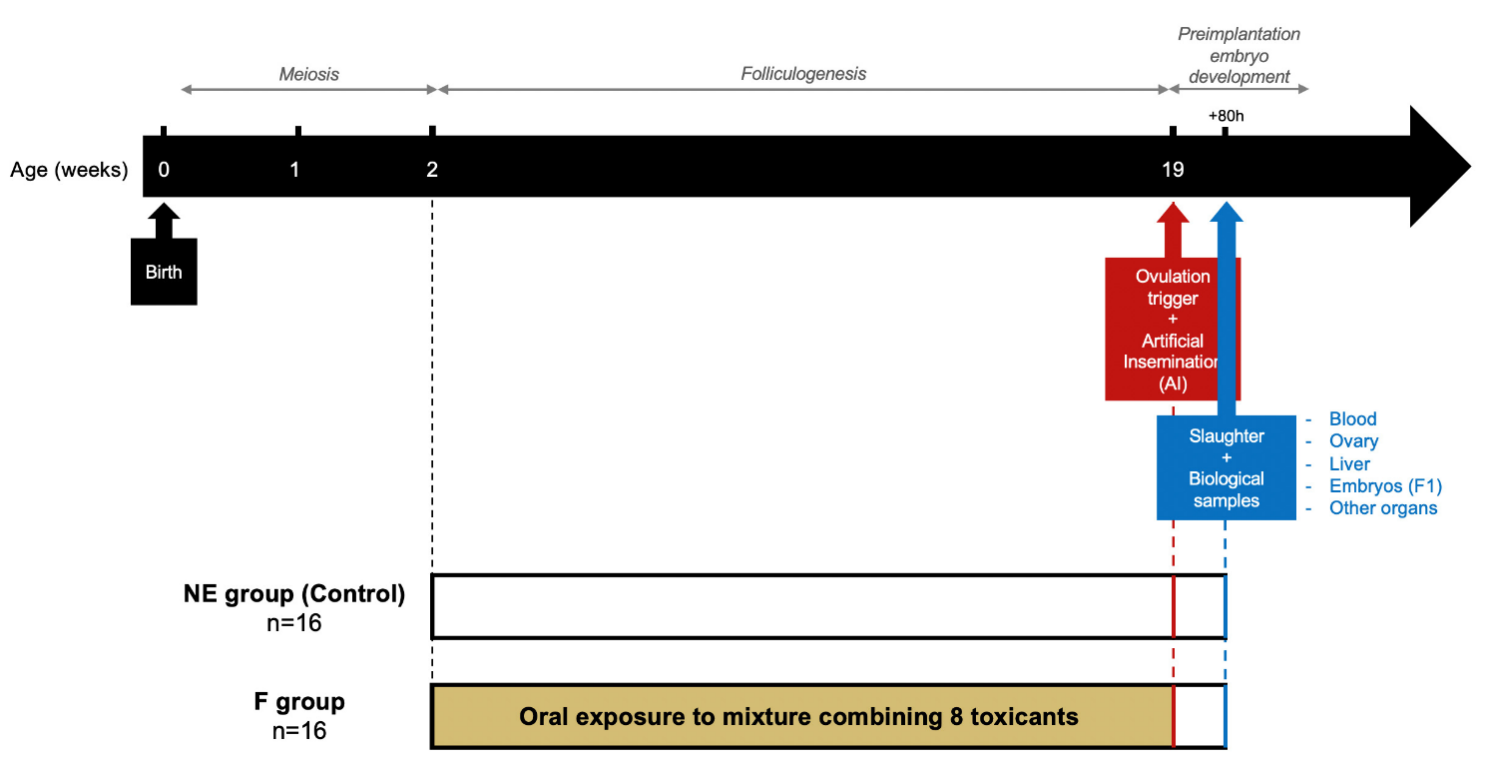
Experimental design. The study includes two groups of female rabbits. Oral and daily exposure lasted 17 weeks, from 2 to 19 postnatal week. The control group (NE group) was exposed to the mixture vehicle (corn oil with 20% or 5% ethanol) while the exposed group (F group) was exposed to the mixture. Females were artificially inseminated with nonexposed semen at sexual maturity (19 weeks) and were sacrificed at 80 h post insemination to recover different biological samples

This study is part of the FEDEXPO project (see project details in [34]). Here, the goal was to evaluate the effects of 17 weeks of exposure to a mixture of human-relevant xenobiotics. The females in the exposed group (F group, *N* = 16) were exposed to the toxicant mixture for 17 weeks, while the control group (NE group, *N* = 16) consisted of females exposed to the solvent mixture only. Oral exposure to the mixture or the vehicle, depending on the group, was performed daily and started at 2 weeks of age, i.e., at the beginning of folliculogenesis in rabbits [36], and ended at 19 weeks of age (Fig. 1). At this time, females were injected with a bolus of GnRH analog (Receptal^®^, MSD Animal Health, Brussels) to trigger ovulation and artificially inseminated with semen from nonexposed males. The slaughter of the animals was performed 80 h after insemination by electronarcosis and bleeding, allowing blood collection. The ovaries were recovered immediately after the bleeding. The liver, thyroid, brain, peri-ovarian adipose tissue, oviducts, uterine horns and preimplantation embryos were also harvested for further investigations. For each group of 16 animals, the exposure start was staggered over 4 weeks per groups of 2 animals (8 repeats i.e. 2 groups starting the exposure on 2 consecutive days per week).

### Toxicant mixture and exposure

The mixture included BDE-47, α-HCB, PFOS, DEHP, PFOA, DDE, β-HCH and BPS. The rationale for this selection has been previously published [34]. Briefly, the eight compounds were chosen according to data taken from the HELIX cohort [38–40] and combined with data from two other cohorts [41, 42]. The goal was to resemble the exposomes of pregnant European women. Toxicants were chosen nature-wise by selecting representatives of major chemical family biomonitored, and concentration-wise by reproducing relevant human serum concentrations in a rabbit model that equalled ten times the 95th (or 90th) percentile (for details, see [34]).

Our team conducted a preliminary pharmacokinetic study to determine the loading and daily maintenance doses of the eight compounds to be simultaneously administered by the oral route in a mixture. The target serum concentrations are detailed in Table 1 [37]. The first-day dosing solution and the maintenance solution were prepared by dissolving the compounds in corn oil (Sigma-Aldrich, St. Louis, MO, USA) containing 20% and 5% ethanol (Sigma-Aldrich, St. Louis, MO, USA), respectively (Table 1). First-day dosing and maintenance solutions were administered orally at 4 and 1 mL/kg, respectively, and the volume was adjusted weekly to the mean bodyweight estimated weekly from the known pattern of growth of the INRA 1777 rabbit line. This protocol allowed us to obtain ratios between the observed and targeted serum concentrations ranging from 0.77 to 1.21 depending on the xenobiotic treatment, as previously described [37].

**Table 1:** Concentrations of the compounds in the exposure solutions required to reach the target concentrations in the rabbit model. The targeted serum concentrations were reached by using a loading dosing solution on the first day and a maintenance solution during the following days. The first-day dosing solution contained the 8 compounds in corn oil containing 20% ethanol and was administered at 4 mL/kg. The maintenance solutions contained the test compounds in corn oil containing 5% ethanol and were administered at 1 mL/kg

Compounds	First-day dosing solution	Maintenance solution	Targeted serum concentration	References for humans 95th or 90th percentiles
BDE-47	2.5 µg/mL	1.1 µg/mL	0.156 ng/mL	[38]
BPS	38.5 µg/mL	122 µg/mL	1 ng/mL	[41]
HCB	46 µg/mL	5.6 µg/mL	2.4 ng/mL	[38]
β-HCH	44 µg/mL	10 µg/mL	4.410 ng/mL	[39]
DDE	287 µg/mL	96 µg/mL	21.18 ng/mL	[38]
PFOA	4.9 µg/mL	10 µg/mL	53 ng/mL	[38]
DEHP	258 µg/mL	770 µg/mL	60 ng/mL**	[42]
PFOS	14 µg/mL	1 µg/mL	200 ng/mL	[38]

Perfluorooctanesulfonic acid (PFOS); perfluorooctanoic acid (PFOA); dichlorodiphenyldichloroethylene (DDE); hexachlorobenzene (HCB); β-hexachlorocyclohexane (β-HCH); 2,2′4,4′-tetrabromodiphenyl ether (BDE-47); di(2-ethylhexyl) phthalate (DEHP); bisphenol S (BPS);

** Targeted MEHP serum concentrations following oral administration of DEHP

### Serum analysis

After collection, the blood samples were left in an upright position for at least 30 min at ambient temperature to allow clot formation. Then, the samples were centrifuged at 3500 rcf for 15 min to recover the supernatant, i.e., the serum. Serum samples were aliquoted and frozen at -20 °C prior to analysis.

Testosterone, dehydroepiandrosterone (DHEA), estrone, estradiol (E2), Δ4-androstenedione, progesterone, and pregnenolone were measured simultaneously by gas chromatography coupled to mass spectrometry according to the protocol described by Giton et al. [43], with minor modifications yet used for the rabbit species [44, 45]. Saline water and twice charcoal dextran-stripped aged female rabbit serum (1/1, v/v) was used as negative and positive quality control standards respectively, and the later as the matrix for calibrators. Limits of quantification and analytical control validation are detailed in a supplemental (Table S1). Luteinizing hormone (LH), follicle stimulating hormone (FSH) and anti-Mullerian hormone (AMH) serum levels were assessed with an Elabscience^®^ Rabbit LH ELISA kit (Houston, TX, USA), an Elabscience^®^ Rabbit FSH ELISA kit (Houston, TX, USA) and a Beckman Coulter AMH Gen II ELISA (Beckman Coulter, Villepinte, France) yet used for the rabbit species [45]. ELISA kits for rabbit LH and FSH dosages both had a sensitivity of 0.94 mIU/mL and detection range between 1.56 and 100 mIU/mL. The AMH ELISA kit reliably detected concentrations as low as 0.08 ng/mL with a 95% probability but the minimum detectable dose with an imprecision rate of 20% is 0.16 ng/mL. Levels over the limit of quantification were obtained for all NE rabbits for DHEA, E2, AMH, and only *n* = 13 NE rabbits for testosterone and progesterone. In the F exposed group, measurements were over the limit for all animals for DHE, AMH while only 15 measures were yield for E2 and 12 for testosterone and progesterone. Serum levels of cholesterol (total and HDL cholesterol), triglycerides, insulin, glucose, aspartate aminotransferase (ASAT) and alanine aminotransferase (ALAT) were measured for *n* = 15 NE animals and *n* = 14 F animals using Beckman Coulter equipment similar to that used in a previous study [46].

### Macroscopic analysis

Ovaries were weighed and checked under a magnifying glass to establish a macroscopic count of corpora lutea (CL), enabling us to check that ovulation did occur. Such parameters were measured for both ovaries in *n* = 11 NE animals and *n* = 12 F animals. The right ovaries were subjected to molecular biology and sectioned into two parts: one frozen in RNAlater (Invitrogen by Thermo Fisher Scientific, MA, USA) and the other snap frozen in liquid nitrogen. The left ovaries were intended for histological and immunohistochemical analysis. The left ovaries were included in cassettes and preserved in formaldehyde solution (4% buffered pH 7) for up to three days before inclusion in paraffin.

### Ovarian histology and immunohistochemistry

Each left ovary was first transversely sectioned in three equal portions. The three portions were embedded closely together in paraffin, with the cutting plane facing down. The sectioning (3 μm) was organized around three sublevels equally distributed over each third of the ovary. This multilevel sectioning method of tri-partitioned blocks was selected because rabbit ovaries are generally larger than those of rodents. This repartition of nine levels across the ovary is considered enough to be representative of the ovary.

#### Hematoxylin and eosin (HE) histology

Deparaffinized and rehydrated ovarian sections were stained with hematoxylin (5 min) and eosin (1 min 20 s) (both from Avantor (VWR), PA, USA) using a TissueTek Prisma Film automated stainer (Sakura FineTek Europe B.V., Villeneuve d’Ascq, France). Whole hematoxylin-eosin-stained slides were digitized with a Pannoramic 250 Flash II digital microscope (3DHISTECH, Budapest, Hungary) equipped with a Zeiss Plan-Apochromat 20X NA 0.8 objective and a CIS VCC-FC60FR19CL 4-megapixel CMOS sensor (unit cell size 5.5 × 5.5 μm) mounted on a 1.6X optical adaptor to achieve a scan resolution of 0.24 μm/pixel in the final image (corresponding to 41.1X magnification at the highest optical resolution in traditional microscopy). The left ovary was analysed through seven complete HE sections among the nine prepared per ovary for 13 ovaries from the NE group (*n* = 91 sections), and 12 ovaries from the F group (*n* = 84 sections). The sections were blindly analysed using SlideViewer software (3DHISTECH, Budapest, Hungary) by one operator. The histological analysis aimed to detect changes in the different populations of follicles potentially caused by the exposure. Analyses were performed using the adjacent section as a reference to avoid double counting the same follicle. On the analysed section, we counted only follicles containing an oocyte whose nucleus and nucleoli appeared clearer than those on the adjacent section. In this way, follicles were counted across the entire section from the left up to the right down. Each follicle was staged as primordial, primary, secondary or antral as previously described [15]. Atretic follicles were counted among secondary and antral follicles according to the criteria of Grzesiak et al. [47] with minor modifications i.e. follicles containing at least 1 (secondary follicles) or 5 (antral follicles) cells exhibiting pyknotic nuclei. The surface area of each section was measured with the annotation tools of the SlideViewer software. For each follicle category, we calculated the density, i.e., the number of follicles per surface unit. We also estimated the total number of follicles per whole ovary. For one ovary, the estimated total count **T** was calculated with the following equation: $T=\frac{n * N}{S}$ (n is the follicle count in S complete sections, N is the total number of 3 μm sections and **S** is the number of complete sections analysed*).* Surface area of antral follicles, along with the surface areas of granulosa and thecal compartment were measured similarly to whole sections area. Values were obtained only for ovaries with antral follicles i.e. *n*=10 NE ovaries and *n* = 11 F ovaries.

#### Cellular proliferation assessment with Ki67 immunohistochemical (IHC) staining

IHC was automated using a Discovery ULTRA (ROCHE Diagnostics, Tucson, USA) staining platform. Ovarian tissue sections were deparaffinized, rehydrated and heated pre-treated at 98 °C using CC1 pre-treatment buffer (pH 9, 32 min; ROCHE Diagnostics, Tucson, USA). The slides were subsequently blocked using peroxidase blocking agent and incubated with anti-rabbit Ki67 (32 min, 1/100 in DAKO Flex Antibody Diluent (Agilent Technologies, CA, USA), clone 8D5, Cell Signaling Technologies, Ozyme, France). The antibody-target complex was then visualized using OmniMap anti-mouse-HRP secondary antibodies (24 min, Ready-to-use, ROCHE Diagnostics, Tucson, USA) and a ChromoMap DAB detection kit (ROCHE Diagnostics, Tucson, USA). The slides were finally counterstained using hematoxylin followed by Bluing reagent (ROCHE Diagnostics, Tucson, USA) and mounted using xylene-based mounting agent (Sakura FineTek Europe B.V., Villeneuve d’Ascq, France). Whole-IHC-stained slides were scanned at high resolution as mentioned above and analysed using Definiens Tissue Studio 4.4.3 (Definiens Developer XD 2.7.0, Munich, Germany). The analysis was performed on 9 sections per ovary for 16 females (*n* = 144 sections per condition). A two-step semiautomated algorithm was used to measure the ratio of Ki67-positive nuclei, and a qualitative analysis of the staining was performed. The first step allowed us to manually define 4 regions of interest (ROIs) in tissue sections, namely whole ovary, medulla, follicles and corpora lutea, by manually outlined the entire ovarian section, all follicles (from primary to antral), or corpora lutea using the tracing tool of the software. The ROI ‘medulla’ was obtained by subtracting the sum of follicles and corpora lutea ROI areas from the whole ovary ROI area. The second step consisted of setting thresholds for immunohistochemistry detection and nuclear size to define a nucleus as marked. These settings relied on a training set of 12 sections chosen randomly and were saved in an analysis profile that was later batch applied to all slides.

#### DNA damage assessment with the TUNEL assay

Nuclei with DNA fragmentation were revealed with a terminal-deoxynucleotidyl transferase-mediated nick end labelling (TUNEL) assay. Deparaffinized and rehydrated ovarian sections were manually treated following the manufacturer’s instructions (In Situ Cell Death Kit TMR Red, ROCHE Diagnostics, Tucson, USA) with the addition of Hoechst nuclear counterstain. Two-channel spectral imaging was performed as previously described [48] using a Pannoramic 250 Flash II whole-slide scanner (3DHISTECH, Budapest, Hungary) equipped with a Zeiss Plan-Apochromat 20X NA 0.8 objective, with appropriate narrow bandpass excitation and emission filters and specific dichroic mirrors (Semrock Inc., Rochester, NY, USA), and with a multichannel solid-state light engine (Lumencor Spectra 7, Lumencor, Inc., Beaverton, OR, USA). Analog to digital image sampling was performed at 8-bit (256 Gy levels, 37 000:1 dynamic range) with a high-resolution scientific complementary metal oxide semiconductor (sCMOS sensor with 2048 × 2048 cells of size 6.5 × 6.5 μm each) Peltier-cooled monochrome camera (PCO.edge 4.2, PCO AG, Kelheim, Germany) to achieve a final scan resolution of 0.32 μm/pixel. Object-oriented whole-slide image analysis was carried out using the Halo^®^ framework version 3.4 (Indica Labs, Albuquerque, New Mexico, USA). The analysis was performed on 3 sections per ovary on 13 F and 12 NE females (*n* = 75 sections in total). Like in the Ki67 analyses, the ROIs (whole ovary, secondary follicles, antral follicles) were delimited manually by one operator blinded to the exposure groups. HE- and Ki67-stained sections were used as a reference for identifying structures. In the Halo^®^ HighPlex algorithm, nuclear contour detection and segmentation were optimized based on the Hoechst counterstain as well as various parameters and morphological features in real-time fine-tuning mode using graphical overlays (signal intensity and contrast, nuclear size and roundness, segmentation aggressiveness). Individual cell objects were then simulated by growing nuclei object boundaries, and the TUNEL signal intensity was detected within cell nuclei objects by thresholding gray-level signal intensities.

### Steroidogenic enzyme gene expression using real-time PCR

Total RNA was extracted from frozen samples via classic phenol/chloroform extraction. RNAs were then suspended in nuclease-free water before their concentration and quality were measured with a NanoDrop^®^ (Thermo Scientific, Waltham, MA, USA) and an Agilent 4200 TapeStation. RNAs were reverse transcribed using a High-Capacity cDNA Reverse Transcription Kit (Applied Biosystems, Courtaboeuf, France). Reverse (R) and forward (F) primers for SYBR Green assays were constructed using the NCBI primer design tool https://www.ncbi.nlm.nih.gov/tools/primer-blast/ (accessed on September 14, 2023) and purchased from Eurogentec (Seraing, Belgium). The sequences are available in Table 2. Amplifications were performed on a Bio-Rad CFX96X™ Real-Time System. The data were recovered using Bio-Rad CFX Manager software (version 3.1.1517.0823). Baseline determination of the mean PCR efficiency and related parameters was performed with LinRegPCR software (version 2021.2). Measurements were obtained for *n* = 14 NE ovaries for *3βhsd*, *5αr2*, *17βhsd4*, *Cyp11a1*, *Cyp17a1*, *Star* and *n* = 13 NE ovaries for *Cyp19a1* versus *n* = 15 F ovaries for *3βhsd*, *5αr2*, *Cyp19a1* and *n* = 16 F ovaries for *17βhsd4*, *Cyp11a1*, *Cyp17a1*, *Star*.

**Table 2:** List of the primers used for RT‒qPCR on rabbit ovaries. All primers used were designed except for the housekeeping gene GAPDH, which was previously described by Mahmoud et al. [49]

Primer	Accession number		Sequence (5’-3’)
*Gadph*	NM_001082253.1	F	AGACACGATGGTGAAGGTCG
		R	TGCCGTGGGTGGAATCATAC
*Cyp19a1*	NM_001170921.2	F	ACACTCATTATCAGCAAGTCCT
		R	AGATCGCCACCATCTGAACA
*3βhsd*	XM_008248712.2	F	GGCCCTTTCCTCTGGGTTTTA
		R	CTTGGTCTTGTTCTGGAGCTTTGAA
*17βhsd4*	XM_008254935.3	F	GGCCTGGCAAATACGCTTT
		R	CATCCTGCTCCAACCTCAAACA
*5αr2*	XM_002709931.4	F	GGATCTAAGGTTTAGCCTGGGTAT
		R	CAGCCCAAGGAAACACAGAGA
*Cyp17a1*	XM_002718585.4	F	TCCCCTGGCTGAAGATTTTTCC
		R	GGTCCTGGCTGGCATTGTTAT
*Cyp11a1*	XM_051834661.1	F	TCACGAGTACTACCGGAGGC
		R	GATGTCGCCTGAGAACACCC
*Star*	XM_017350353.2	F	TAGCGACGTTCAAGCTGTGT
		R	TAGAGAGTCTCCTCCAGCCG

### Statistical analysis

Statistical analyses were performed using GraphPad Prism for Windows v9.4.1 (GraphPad Software, San Diego, CA, USA). An unpaired t test was used to compare the control and exposed groups if the data sets followed a normal distribution. In that respect, Shapiro–Wilk tests were performed beforehand. Otherwise, the data were analysed via the nonparametric Mann‒Whitney test. A p value less than 0.05 was considered to indicate statistical significance. The results from the weight follow-up were analysed via two-way ANOVA to evaluate the interaction between age and exposure factors.

## Results

### A chronic exposure to a mixture of environmental toxicants does not impact the growth of females but does disrupt serum cholesterol levels

**Fig. 2:**
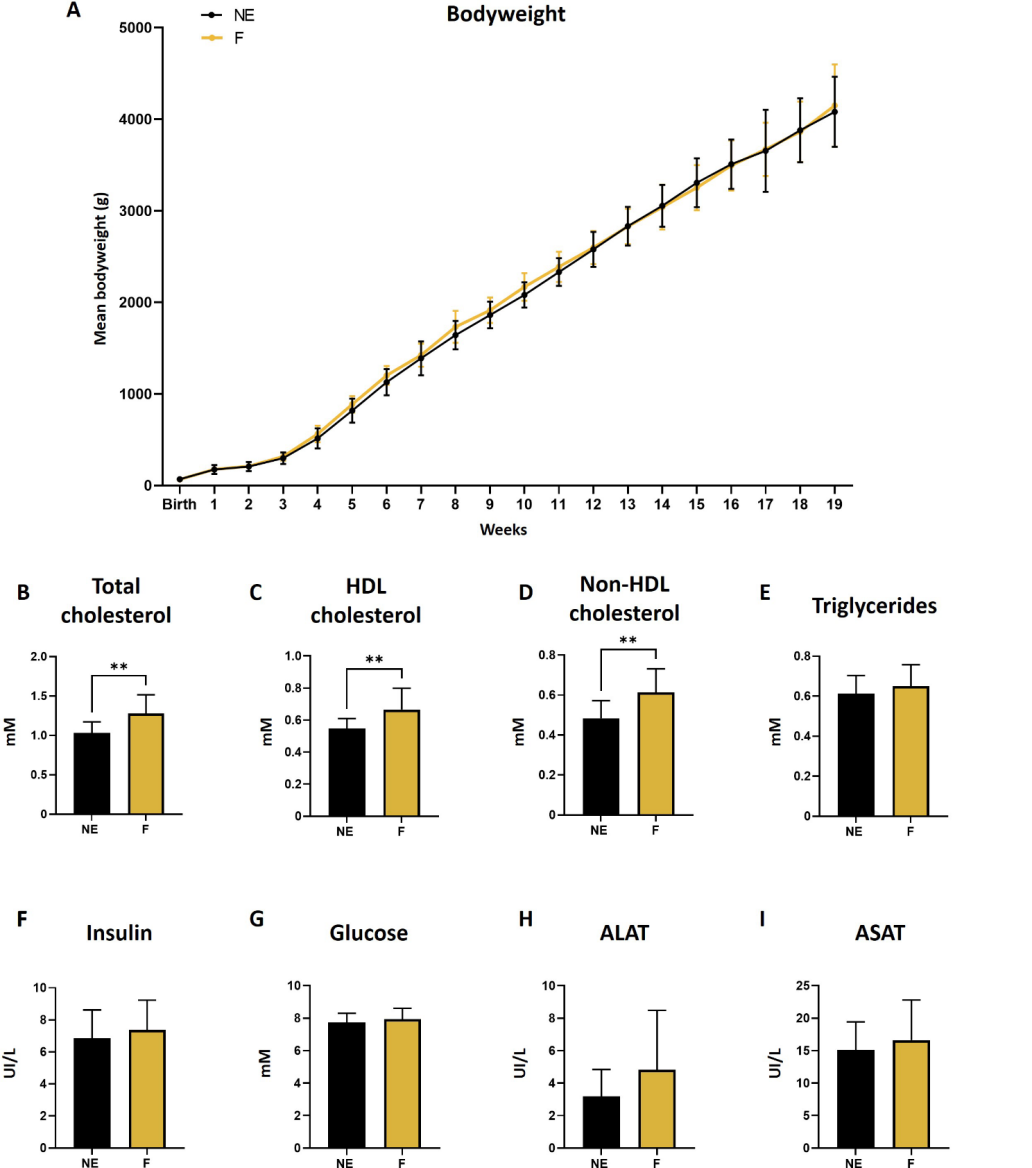
Effect of a folliculogenesis exposure on bodyweight and serum biochemistry at adulthood. **(A)** Bodyweight was weekly measured from birth to 19 weeks (*n* = 16 for both conditions). All biochemical parameters, i.e., serum concentrations of **(B)** total cholesterol, **(C)** HDL cholesterol, **(E)** triglycerides, **(F)** insulin, **(G)** glucose, **(H)** alanine aminotransferase and **(I)** aspartate aminotransferase were measured except for **(D)** non-HDL cholesterol which was calculated using the following equation: non-HDL cholesterol = (Total cholesterol) − (HDL cholesterol). Data are presented as mean ± SD for F (mixture-exposed, *n = 13–14*) and NE (control, *n = 12–15*) groups. **: *p* < 0.01 for F vs. NE

Bodyweight did not differ between the two groups during the experiment (Fig. 2A). Classic biochemical analysis of the serum was also performed to assess the overall health of the females (Fig. 2B-I). Most of the serum parameters, i.e., triglyceride, insulin, glucose, alanine aminotransferase, and aspartate aminotransferase levels, did not significantly differ. However, cholesterol levels, whether it was total, high-density lipoprotein (HDL) or non-HDL cholesterol, were increased in exposed females (Fig. 2B-D).

### A chronic exposure to a mixture of environmental toxicants increases the populations of antral and atretic follicles without impacting cell proliferation, DNA fragmentation or the ovarian reserve of primordial follicles

**Fig. 3:**
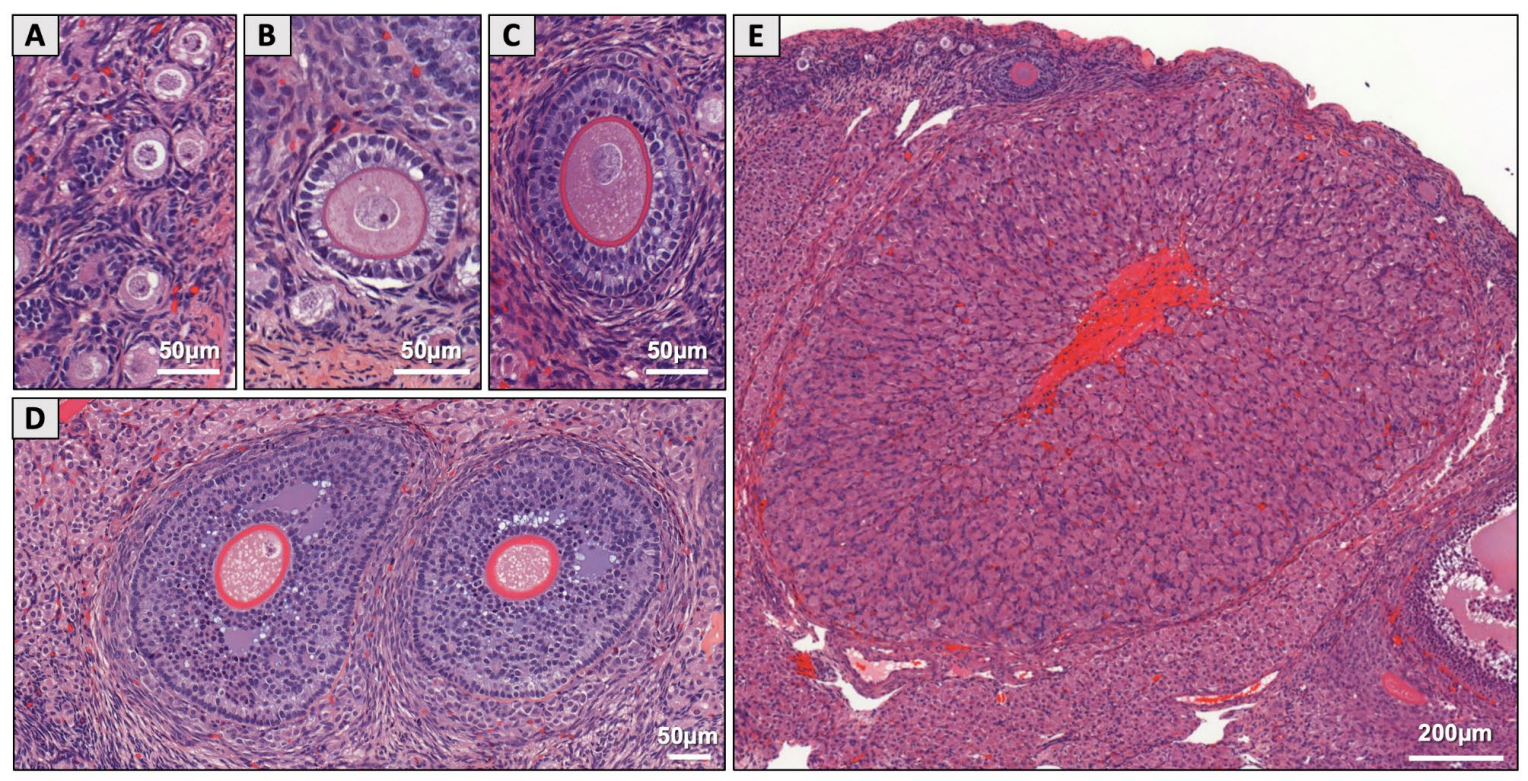
Representative images of HE-stained ovarian section. HE-stained ovarian section allowed to distinguish **(A)** primordial, **(B)** primary, **(C)** secondary, **(D)** antral follicles and **(E)** corpora lutea

**Fig. 4:**
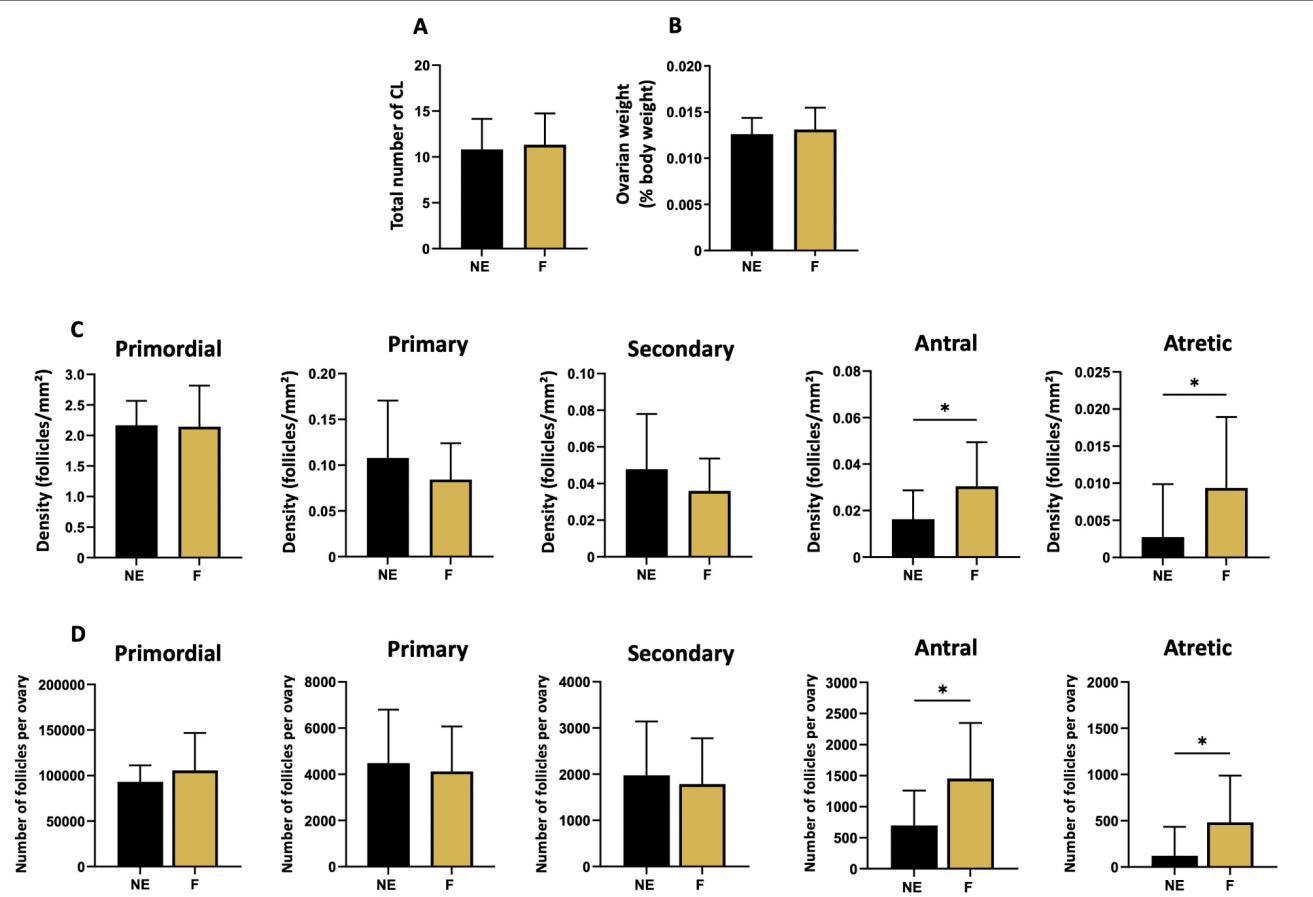
Effect of a folliculogenesis exposure on ovarian parameters assessed through macroscopic and microscopic measurements. **(A)** CL number and **(B)** ovarian weight were measured at sacrifice (*n* = 12 for F (mixture-exposed) and *n* = 11 for NE (control). Histological analysis of HE-stained sections reported the density of follicles **(C)** at each stage based on 7 complete sections. The results were also presented as estimated total count in whole ovary **(D)** for F (*n = 12*) and NE (*n = 13*) groups. Data are presented as mean ± SD. *: *p* < 0.05 for F vs. NE

HE staining allowed the assessment of the different follicular stages and the CLs on the ovarian sections (Fig. 3). Macroscopic analysis of the two ovaries (*n* = 24 ovaries per condition) revealed a similar number of CLs in both groups (Fig. 4A) and a similar weight (Fig. 4B). Histological evaluation of HE sections revealed no significant difference between exposed and control ovaries in terms of the density of follicles at preantral stages, i.e., primordial, primary and secondary follicles (Fig. 4C). The mean density for the primordial stage was 2.23 ± 0.43 follicles/mm² for the control NE group versus 2.33 ± 0.96 follicles/mm² for the exposed F group (NS). For the primary and secondary stages, the results were 0.11 ± 0.06 and 0.05 ± 0.02 follicles/mm², respectively, in the NE ovaries versus 0.09 ± 0.05 and 0.05 ± 0.03 follicles/mm², respectively, in the F ovaries (NS). However, analysis revealed an increased density of antral follicles in the exposed group (0.030 ± 0.02 in F versus 0.017 ± 0.01 mm² in NE, *p* = 0.04; Fig. 4C).

**Fig. 5:**
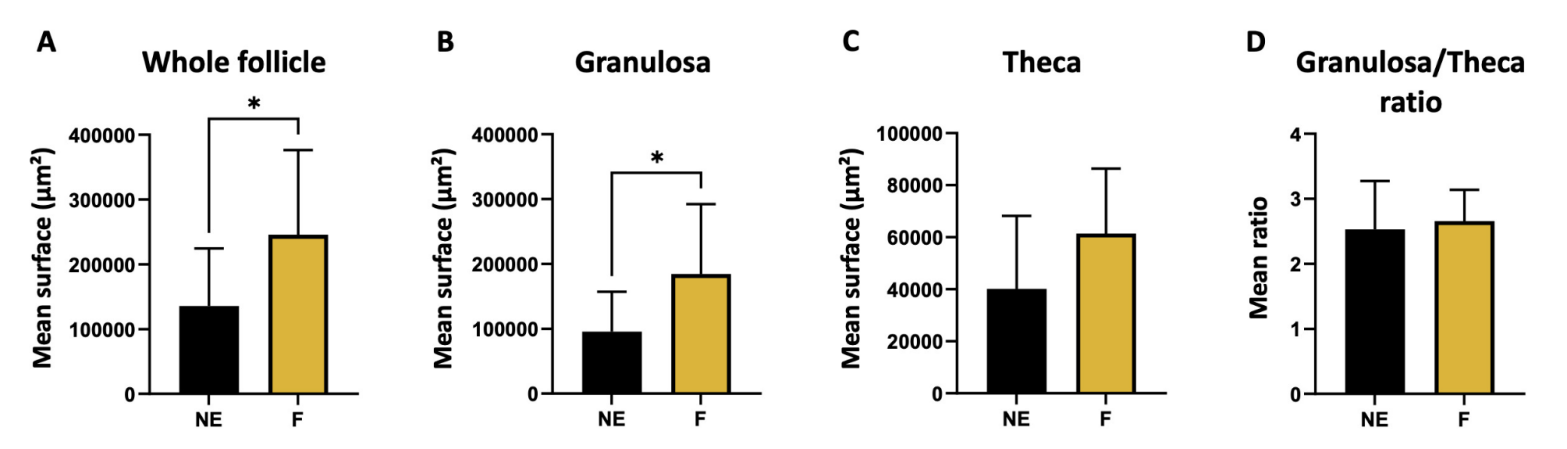
Effect of a folliculogenesis exposure on surfaces of antral follicles. Histological analysis of HE-stained ovarian sections provided the **(A)** mean surface of antral follicles with detailed measurements of **(B)** granulosa, **(C)** theca surface and **(D)** their ratio. Data are presented as mean ± SD for F (mixture-exposed, *n* = 10) and NE (control, *n* = 11) groups. *: *p* < 0.05 for F vs. NE

Similarly, the density of atretic follicles was significantly increased in the exposed group than in the control group (*p* = 0.03; Fig. 4C). Specifically, F ovaries displayed a mean density of 0.009 ± 0.009 atretic follicles/mm² versus 0.0027 ± 0.007 in NE ovaries. The follicle counts also served to estimate follicular populations throughout the ovary (Fig. 4D). The results showed significantly greater counts of antral follicles (*p* = 0.02) and atretic follicles (*p* = 0.03). Furthermore, when assessing the size of the antral follicles, we observed that the antral follicles of the exposed females were significantly larger than those of the controls (0.25 ± 0.14 mm² in F versus 0.14 ± 0.089 mm² in NE, *p* = 0.03; Fig. 5A). The mean granulosa area was also significantly greater in F antral follicles than in NE follicles (*p* = 0.03; Fig. 5B). However, the theca surface area remained similar under both conditions, as did the ratio of granulosa/theca area (Fig. 5C-D).

**Fig. 6:**
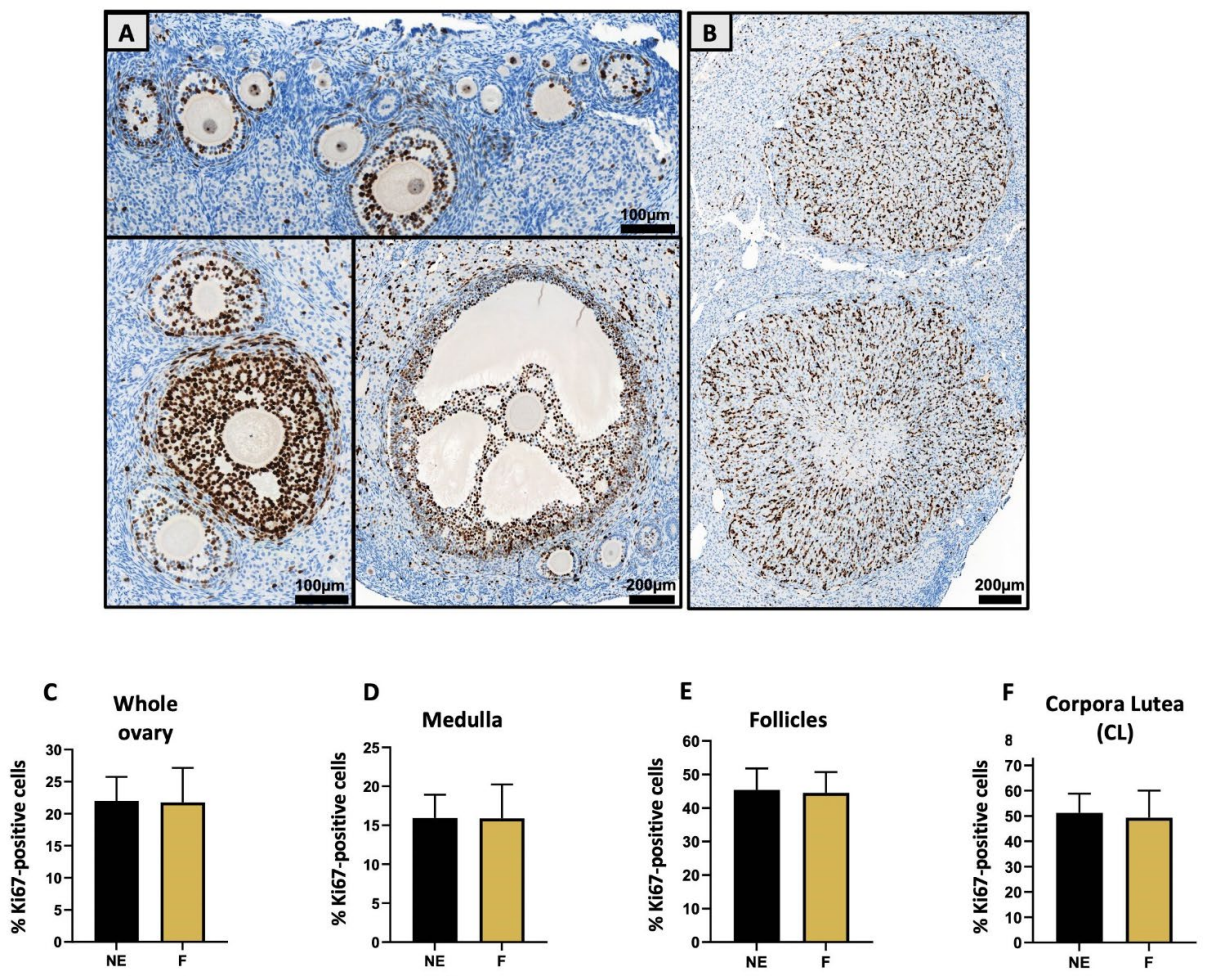
Effect of a folliculogenesis exposure on cellular proliferation in ovaries assessed via Ki67 immunostaining. Ki67-positive nuclei are stained in brown as shown in **(A)** follicles and **(B)** corpora lutea zooms. Bar plots detail the percentages of Ki67-positive cells in **(C)** whole ovaries, **(D)** medulla, **(E)** follicles and **(F)** corpora lutea for F (mixture-exposed) and NE (control) female ovaries. Data are presented as mean ± SD for F (mixture-exposed, *n* = 16) and NE (control, *n* = 16) groups

Histological analyses were completed with immunohistochemistry to study cellular proliferation and DNA fragmentation. Cellular proliferation assessment via Ki67 staining relied on the analysis of Ki67-positive nuclei stained brown among the different ovarian elements (Fig. 6A-B). The two groups showed very similar percentages of Ki67-positive nuclei regardless of the ROI studied. When working with the whole ovary, 21.9 ± 3.8% of cells in the control group had Ki67-positive nuclei, while 21.7 ± 5.4% of cells in the exposed group had Ki67-positive nuclei (Fig. 6C). Similarly, the percentages of Ki67-positive nuclei in the medulla, follicles and CL were similar in both groups (NS; Fig. 6D-F).

**Fig. 7:**
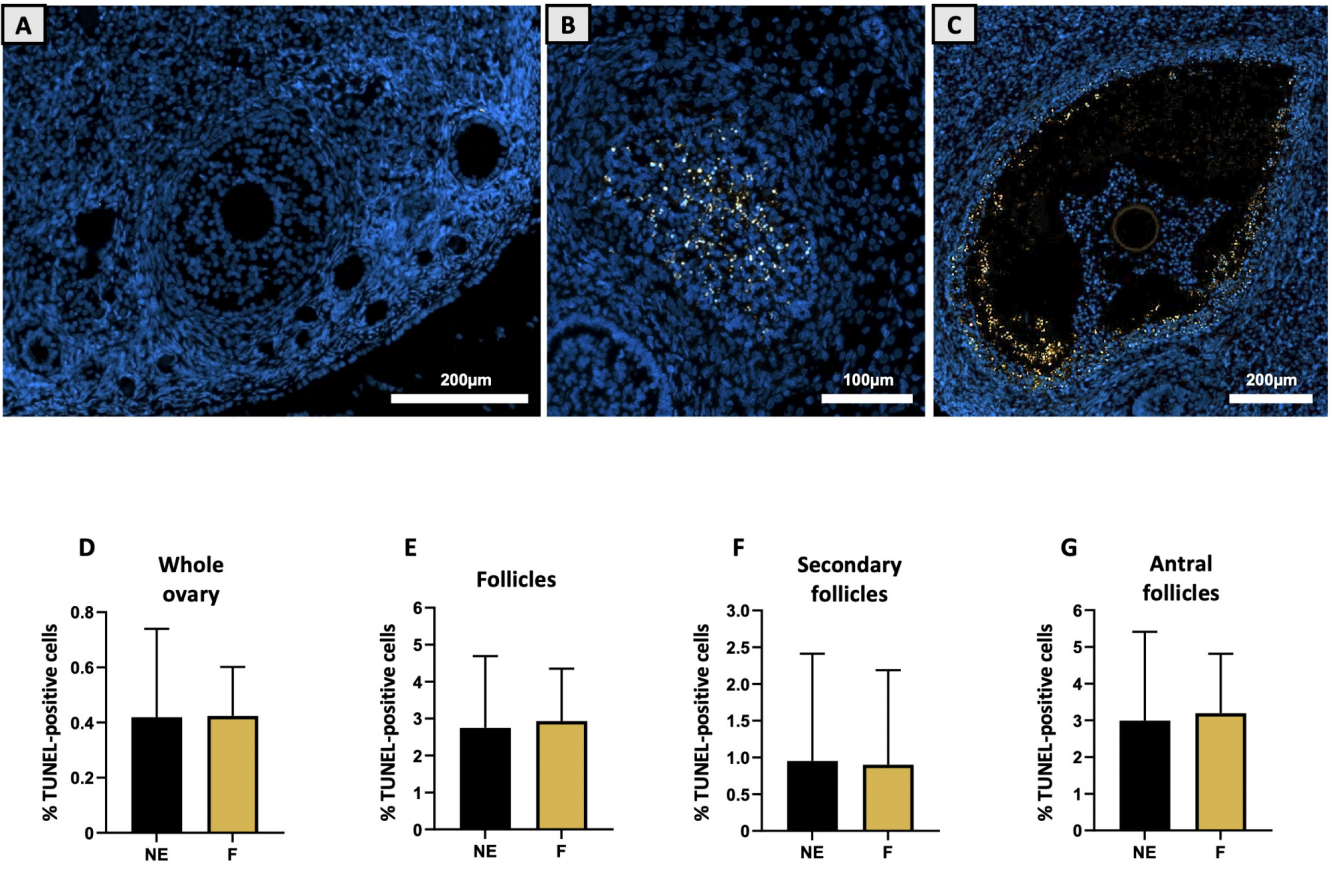
Effect of a folliculogenesis exposure on DNA fragmentation in ovaries assessed via TUNEL assay. DNA fragmentation is evidenced in nuclei colocalizing Hoechst (blue) and TUNEL (orange) marking. TUNEL marking was punctual in ovarian sections in both groups, primarily targeting secondary and antral follicles as shown in **(A-C)** zooms. Bar plots detail the percentages of TUNEL-positive cells in **(D)** whole ovaries, **(E)** follicles including **(F)** secondary and **(G)** antral stages. Data are presented as mean ± SD for F (mixture-exposed, *n* = 13) and NE (control, *n* = 12) groups

DNA fragmentation was revealed by visualizing both Hoechst and TUNEL staining (Fig. 7A-C). Only secondary and antral follicles were analysed via TUNEL staining since they are the main targets of TUNEL staining. No fluorescence was detected in the medulla or in the CL. When TUNEL staining was performed on whole ovarian sections, no significant difference was detected between the two groups (Fig. 7D). The percentage of TUNEL-positive nuclei in the control group was 0.44 ± 0.32%, whereas it was 0.42 ± 0.18% in the F group (NS). The same conclusions were obtained for the follicles (Fig. 7E), even when the secondary and antral follicles were studied separately (Fig. 7F-G). Interestingly, while exposed antral follicles displayed an increased size when measured on HE sections compared to control, the mean number of cells (Hoechst positive nuclei) per antral follicles were remarkably similar between the groups (1566 ± 240 for NE vs. 1529 ± 212 for F; NS).

### Folliculogenesis exposure disrupts steroidogenesis

**Fig. 8:**
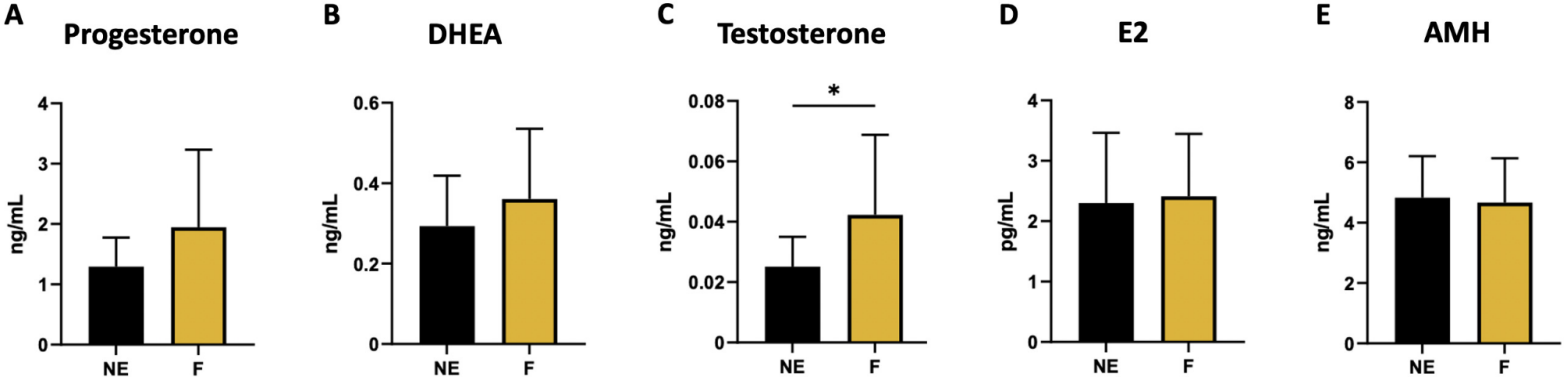
Effect of a folliculogenesis exposure on serum hormone concentrations. Bar plots present concentrations of **(A)** progesterone, **(B)** dehydroepiandrosterone, **(C)** testosterone, **(D)** estradiol and **(E)** Anti-Mullerian Hormone. Data are presented as mean ± SD for F (mixture-exposed, *n = 12–16*) and NE (control, *n = 13–16*) groups. *: *p* < 0.05 for F vs. NE

The measurements of LH, FSH, androstenedione, pregnenolone and estrone yielded values under the limit of quantification that are not presented. The mean (± SD) serum concentrations of AMH, progesterone, DHEA, testosterone, and E2 are presented in Fig. 8. Statistical analysis revealed a significant increase in the testosterone concentration in the serum of exposed females (Fig. 8C).

**Fig. 9:**
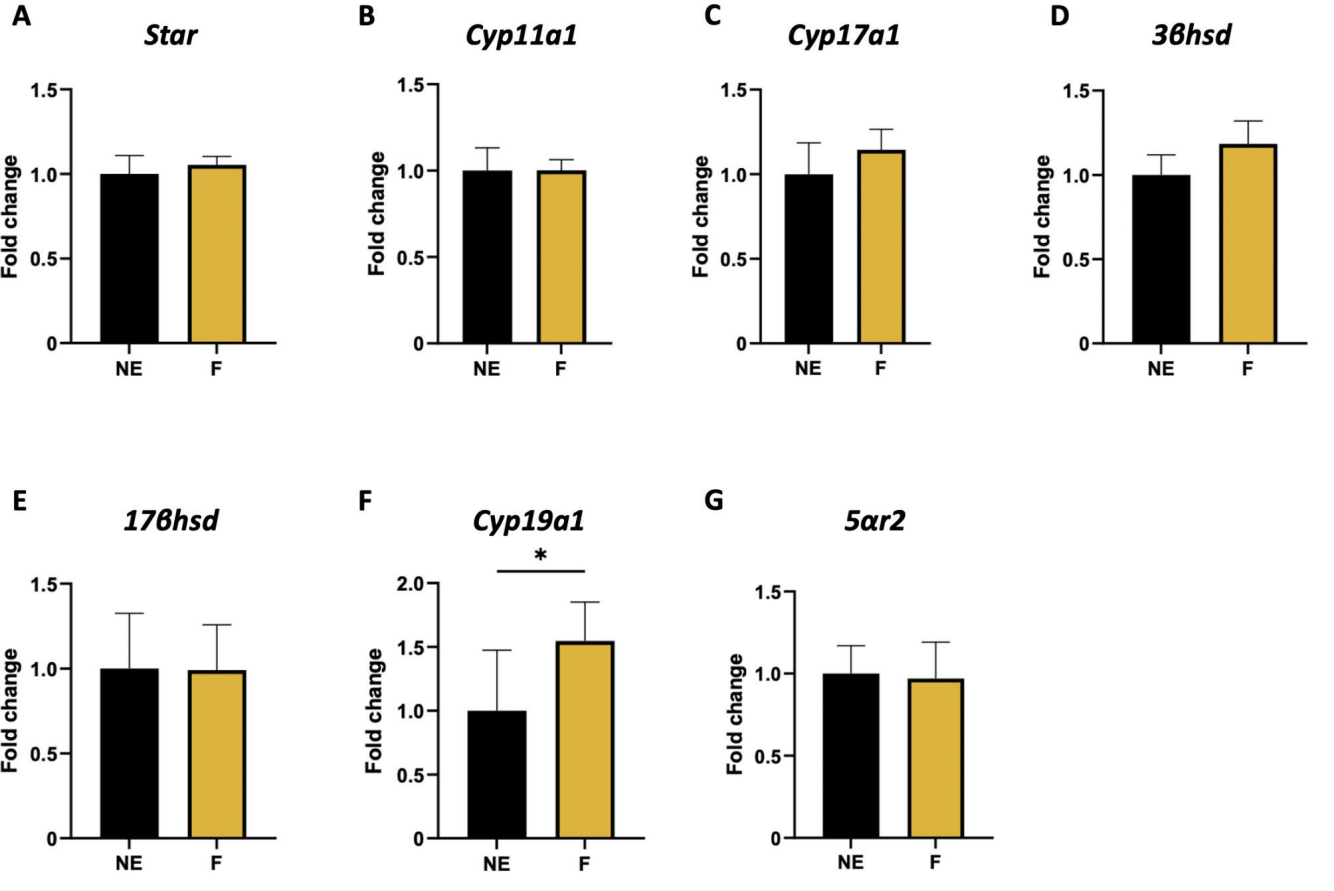
Effect of a folliculogenesis exposure on gene expression of steroidogenesis enzymes tested by qRT‒PCR. Bar plots present fold change for **(A)***Star*, **(B)***Cyp11a1*, **(C)***Cyp17a1*, **(D)***3βhsd*, **(E)***17βhsd*, **(F)***Cyp19a1*, **(G)** 5*αr2* genes. Data are presented as mean ± SEM for F (mixture-exposed, *n = 15–16*) and NE (control, *n = 13–14*) groups. *: *p* < 0.05 for F vs. NE

We subsequently measured the expression of several genes encoding key steroidogenesis enzymes. Exposure did not alter the expression of the *Star* or *Cyp11a1* genes (Fig. 9A-B), which encode respectively for the steroidogenic acute regulatory protein (StAR) and the cholesterol side-chain cleavage enzyme (cytochrome P450 scc) respectively. These enzymes play crucial roles at the beginning of steroidogenesis by mediating cholesterol transfer and converting the latter to pregnenolone. Similarly, intermediate enzymes such as cytochrome P450 17A1 and dehydrogenases (Fig. 9C-E) were not different in terms of gene expression. *Cyp19a1* expression was significantly greater in F ovaries than in control ovaries. This gene encodes an enzyme responsible for androgen-to-estrogen conversion, aromatase. Finally, the expression of the *5αr2* gene, which encodes steroid-5-alpha reductase, remained similar in both groups. The latter enzyme is not involved in steroidogenesis but is important for testosterone homeostasis because it converts testosterone into dihydrotestosterone (DHT), a more potent form of testosterone.

## Discussion

Folliculogenesis is a crucial process for female reproduction, and the study of the impacts of exposure to xenobiotics during this process seems relevant. To our knowledge, the present article is the first to report the in vivo effects of realistic exposure to a mixture of environmental contaminants on ovarian function. We showed that combined 17 weeks of exposure to 8 compounds at serum levels relevant for women induced slight alterations in some parameters of folliculogenesis without affecting the ovarian reserve. Firstly, analysis of ovarian sections revealed no change in preantral follicles populations but an increase in the density, total number and size of antral follicles. Secondly, exposed animals displayed more atretic follicles but no changes in cell proliferation or DNA fragmentation. Thirdly, serum analysis showed no effect on the levels of most steroids analysed, with the exception of a higher testosterone levels in exposed females, while the expression of genes encoding key steroidogenic enzymes were similar except for an increase in *Cyp19a1* gene expression.

The first objective of our study was to investigate the effects of the mixture on the dynamic of folliculogenesis. Evaluations of follicular populations, especially primordial and antral pools, are almost mandatory steps in ovarian toxicological studies and provide two important pieces of information regarding the dynamics of folliculogenesis. The primordial pool represents the foundation of follicular populations and is therefore a determining factor for the quality and duration of fertility [50]. On the other hand, the antral follicle count (AFC) reflects active and developed follicles that can potentially be ovulated. During this study, ovarian histological analysis was assessed 80 h after ovulation induction and artificial insemination. We did not reveal differences in terms of density or total number of primordial, primary or secondary follicles nor corpora lutea while the density and estimated total number of antral follicles were significantly greater in the exposed ovaries. Similar results for primordial and antral follicles were reported by Lefèvre et al. [12], who studied the ovaries of rats fed a diet supplemented with a mixture of brominated flame retardants (BFRs) before mating and during gestation. Lea et al. did not observe differences in the number of antral follicles in the fetal ovaries of ewes exposed to sludge fertilizer during gestation; however, they reported a greater number of primordial follicles in the group exposed during late gestation [18]. Other studies reported no significant changes in either parameter following perinatal exposure to various mixtures [21, 22]. One last study reported a decrease in the number of antral follicles in ovaries from mice exposed from conception to the adult stage to a mixture of phthalates and alkylphenols [51]. The results mentioned above vary unsurprisingly considering the differences in terms of species, exposure timing, mixture composition and concentrations, making any comparison difficult. We emphasize that one strength of our study is the use of a solid methodology for ovarian histology analysis, as indicated by the number of experiments, animals, ovarian sections per animal and the blindness of the evaluation. It is quite reassuring to note that the current exposure to a mixture of 8 toxicants usually found in women of child-bearing age does not alter the stock of primordial follicles. Alterations in the primordial follicle stock can induce premature ovarian insufficiency during female life, with significant consequences for women’s reproductive health and quality of life [52]. Our data are consistent with a previous study by our team on the ovarian effects of a mixture of pesticides at low doses using a mouse model [15]. Any discrepancies with other studies in the scientific literature are probably linked to the use of high doses. For example, a study carried out by Johansson et al. [21] demonstrated that perinatal exposure of rat pups to an androgenic mixture induced a significant reduction in primordial follicles; however, the doses used were 100 to 450 times greater than what is considered high human exposure. To our knowledge, there are no animal data demonstrating the harmful effects of exposure to a mixture of xenobiotics at human-relevant doses on the ovarian primordial follicle reserve. Given that our model was not designed to take into account exposure during the initiation of meiosis in the female germ line or the assembly of primordial follicles (corresponding to maternal exposure during pregnancy in humans), we cannot exclude any effect of our mixture of toxicants on the fetal ovary and its primordial follicles.

To further investigate the increase in antral follicles density and estimated total number, we analyzed cell proliferation by Ki67 staining. Strikingly, we did not observe difference regarding cell proliferation in follicles between control and exposed rabbits. One major limit of this analysis is the inclusion of all follicle types, from primary to antral, within the follicle ROI. Even though primary follicles were poorly marked by Ki67, secondary and antral follicles were both subjected to a relatively important staining. Such analysis needs to be reiterated distinguishing secondary from antral follicles to assess if the increase in antral follicles number is associated with an increased cell proliferation in secondary follicles in exposed ovaries. Furthermore, the size of antral follicles is significantly higher in exposed rabbits due to a larger granulosa compartment. Nonetheless, caution is warranted since the measurements of granulosa compartment included antral cavities. We cannot deny the contribution of antrum in the increased granulosa compartment since TUNEL data showed that antral follicles had the same number of cells in both groups. Granulosa cells express AMH which serum concentration is considered a reliable marker of ovarian reserve in humans and domestic animals [53–58] and is generally closely positively associated with the ultrasound AFC i.e. antral follicles. However, in the rabbit, no correlation between serum AMH and AFC was evidenced in a recent study [59] where AMH was correlated with secondary follicles only. Therefore, the lack of difference in the proportion/number of secondary follicles between exposed and control groups can explain their similar serum AMH levels.

Apart from studying the impact of the mixture on follicular populations, we looked at its impacts on follicular cell death using both visual and TUNEL analysis. Haematoxylin and eosin analysis demonstrated a significant increase in the density/total number of antral atretic follicles in the exposed ovaries. Using the same approach, Lea et al. reported an increase in atretic primordial follicles in the fetal ovary of ewes whose mothers were exposed to sewage sludge-treated pastures during the gestation period (offspring follicle formation), but not in follicles at preantral and antral stages combined [18]. Two studies did not show significant differences in follicular (stages not specified) atresia when adult female rats were exposed to a mixture of BFRs during gestation [12] or perinatally to a high-dose mixture of endocrine disruptors [21], and two other studies did not investigate atretic follicles [22, 51]. Our TUNEL assay did not reveal differences in DNA fragmentation, suggesting that the occurrence of nuclear apoptosis was similar in both groups while the number of atretic follicles increased in exposed ovaries. These results are not contradictory since nuclear apoptosis is not the only mechanism leading to follicular atresia. Although the literature is still scarce [60], autophagy and necrosis appear to be alternative pathways of nuclear cell death in ovarian follicles. In mice, necrosis appears to be predominant for small follicles [60], which could explain the lack of TUNEL labelling in our early-stage follicles if it is similar in rabbit. In larger follicles, all three cell death pathways appeared to occur in equal proportions in the mouse model [61]. Leopardo et al. demonstrated that autophagy is one mechanism of cell death in atretic follicles of hares ovary [62]. The increase in atretic follicles in our exposed females could therefore be due to autophagy in granulosa cells and/or mitochondrial apoptosis that is also a cell death pathway for granulosa cells [63]. In summary, the current findings indicate that a chronic human-relevant exposure to a mixture of toxicants through folliculogenesis promotes the late follicle development, as evidenced by higher density, estimated total number and size of antral follicles in exposed animals. However, this is occurring alongside the increase of atretic antral follicles without affecting the number of CL. These observations suggest that the supernumerary antral follicles in exposed ovaries do not reach ovulation, potentially due to atresia for which the exact mechanism have not been elucidated in this work. Interestingly, another research group also showed a stimulation of late follicular development with increased incidence of atresia after a neonatal exposure to BPA [64].

Finally, the last aim of our study tackled the effect of our mixture exposure on the sex hormone profile. Some hormonal dosages yielded values under the LOQ for the gonadotropins and some steroids. In most cases, the low levels are probably a consequence of the timing of blood collection. LH and FSH serum levels 80 h after ovulation trigger are supposed to be very low since the gonadotropins peak in the first hours following post coitus [65]. Resources providing levels of all steroids throughout pregnancy in rabbit are rare, or non-existent for the case of pregnenolone and androstenedione. One study reported a peak in androstenedione peripheral level occurring at 4 h post ovulation trigger and low but measurable dosages in the 9 h following GnRH stimulus [66]. We found only one study providing data for estrone plasma level in pregnant rabbits [67]. On the day 3 of pregnancy, the authors reported values largely included in our detection range, i.e. about 20pg/mL of estrone in arterial plasma. The reliability of these reported results in pregnant rabbits can be questioned due to factors such as the age of the rabbits (that was not specified) and the utilization of radioimmunoassay and its limitations, including cross-reactivity with other steroids and reduced accuracy compared to GC-MS. GC-MS measurements revealed a significant increase in the serum testosterone concentration in the exposed group. Although it is circulating and not intrafollicular (could be less informative when examining oocyte and ovarian issues [68, 69]), the testosterone concentration is a relevant reproductive endpoint and plays an important role in ovarian, uterine [70–72] and oocyte health [73]. An increase in testosterone levels after exposure to a xenobiotic mixture has been reported in vivo in previous studies [12, 18] and in vitro in porcine granulosa and thecal cells cocultured with a mixture of PBDEs [26]. In the latter study, the results differed when DDT and DDE were present in the mixture, clearly illustrating the complexity of mixing molecules, even within the same chemical family. Different compounds isolated from our mixture have also been associated with increased testosterone levels in the literature, including PFOA [74], DEHP [75] and BDE-47 [76]. In these studies, as in our study, adrenal participation has not been investigated, although it could have provided valuable information given the role of adrenals in steroid homeostasis [77, 78]. Mechanistically, an increase in testosterone can be associated with different parameters, such as cholesterol and precursor hormone levels, as well as with qRT‒PCR results for steroidogenesis enzymes. Although we detected an increase in the serum cholesterol concentration, no changes were observed in *Cyp11a1* and *Star* gene expression, both encoding for enzymes important in the first step of steroidogenesis and cholesterol absorption. Lefevre et al. highlighted that *Cyp17a1* was downregulated in the ovaries of rat dams exposed to the highest dose of BFRs, but we did not reach a similar conclusion via qRT‒PCR in rabbits. Our analysis revealed an increase in the expression of the gene encoding the aromatase enzyme in the ovary. Patiño-García et al. reported a decrease in relative *Cyp19a1* mRNA levels in mice prenatally and postnatally exposed to a mixture of phthalates and alkylphenols (including DEHP) [50]. On the other hand, compounds such as PFOA and BDE-47 have occasionally been associated with increased expression of *Cyp19a1* [74, 79] alongside disturbances in estrogen levels [74] and activity [79].

Although exposed animals showed increased testosterone levels and *Cyp19a1* expression in ovaries, we demonstrated no difference in the serum estradiol concentration. We hypothesized that this higher gene expression of *Cyp19a1* may be related to both the ovarian response to increased testosterone production and an endocrine disrupting effect of the mixture on aromatase activity, which would affect estradiol secretion. The larger size of the granulosa compartment in antral follicles may reflect a compensatory mechanism for this decreased aromatase activity. We cannot confirm this hypothesis since our study did not include an evaluation of aromatase activity. Furthermore, if confirmed, enzymatic inhibition could be linked to a direct effect of the mixture or indirectly via an increase in testosterone secretion since it has been demonstrated in vitro that testosterone decreases aromatase activity in uncultured human granulosa cells in a dose-dependent manner [80].

Finally, the biochemical results opened another avenue of investigation. Significantly higher cholesterol levels were measured in the serum of exposed females, although their bodyweight remained the same as that of the control group. Few of the compounds studied are known (BPS, DDE [81], DEHP, or PFOA [82]) or suspected obesogens but the present study was not designed to investigate metabolic disruption in depth. It remains a relevant question to study in the future, especially since metabolism and reproductive functions are closely linked. Although some phenotypic changes observed in exposed females, such as elevated testosterone levels and antral follicle numbers or dyslipidaemia, may be reminiscent of a PCOS-like phenotype, their similar weight and larger antral follicles do not correspond to a typical PCOS phenotype. Furthermore, according to Jakimiuk et al., *Star*, *Cyp11a* and *Cyp17* are overexpressed in PCOS follicles [83], and the aromatase gene is under expressed in PCOS-granulosa cells [84], which is the opposite of our findings.

Gathering our ovarian results, we can attempt to draw a cascade of events induced by our mixture of 8 xenobiotics. The exposure does not affect folliculogenesis until the antral follicle stage, preserving the ovarian reserve of primordial follicles and maintaining serum AMH levels since secondary follicles are the main contributors to serum AMH in the rabbit species [59]. AMH is a well-known regulator of the initiation of folliculogenesis from the primordial pool, we can postulate that this pool is preserved from any effect of the mixture: neither a direct cytoxic effect nor an indirect depletion through AMH regulation. From the antral stage onward, we speculate that the mixture of xenobiotics induces an endocrine disrupting effect on Cyp19a1 enzymatic activity, whose mechanism is not elucidated, leading to increased serum testosterone levels. To cope with the lower ability to convert testosterone to estradiol, granulosa cells would overexpress the *Cyp19a1* gene allowing to maintain normal serum estradiol levels. A compensatory response at antral stage could also explain the development of more and bigger antral follicles with a preferential development of the granulosa compartment (including the antral cavity). However, these more numerous antral follicles in exposed ovaries undergo more atresia, the mechanism of which is not nuclear apoptosis according to our TUNEL results. Further studies are needed to identify its exact mechanism and evaluate whether or not it is consecutive to an endocrine disruption. This atresia would maintain the same number of pre-ovulatory follicles, explaining why we observed, 80 h post ovulation, equivalent numbers of corpora lutei, no difference in serum progesterone and the same number of collected embryos (data not shown). The question of whether the quality of these embryos is affected, due to oocyte-driven alterations post exposure to the mixture, is currently under investigation within the FEDEXPO project.

Our study presents some limits. Rabbit are reflex ovulators therefore the mechanisms inducing ovulation post-coitus, although not fully elucidated [85], are different from cycling species like Humans. We also did not evaluate the effects of the mixture on adult folliculogenesis i.e. long after the end of exposure since our window of exposure started once the primordial follicle pool is assembled and ended at sexual maturity (equivalent to post pubertal stage of a young woman). Therefore, complementary studies are needed to confirm these results. Lastly, our experimental design includes a solvent-exposed control. Even though it is a common practice in order to reduce animals number, it constitutes a limit compared to a true negative control i.e. non-exposed to vehicle and toxicants since it prevents to fully isolate and understand the vehicle’s influence and its sole impacts on evaluated parameters.

## Conclusion

In conclusion, our data show that a complex woman-relevant exposure to environmental chemicals during folliculogenesis induced alterations of final folliculogenesis and ovarian steroidogenesis in the rabbit species. As show for some isolated xenobiotics, the mixture resulted in alterations of aromatase expression, a key ovarian steroidogenic enzyme, associated with increased testosterone in blood i.e. an endocrine disrupting effect. Furthermore, the xenobiotics mixture induced a cytotoxic effect on antral follicles. Importantly, no impact on the primordial follicles pool nor ovulation capacities were shown. As far as we know, using a mixture of 8 xenobiotics from 5 different chemical families is unique regarding xenobiotics impacts on mammalian female reproduction. The nuanced effects observed illustrate the difficulty to decipher how the real-life, multiple, low-doses chemical environment of women could affect their reproductive potential. Such strategy using in vivo exposure to complex mixtures is necessary to assess other reproductive endpoints, such as development and implantation of the embryo, but also the health of future descendants.

### Electronic supplementary material

Below is the link to the electronic supplementary material.

Supplementary Material 1

## Data Availability

The data sets supporting the conclusions are not included in the article but are available from the corresponding author upon reasonable request.
