# Supplementary material for: Folliculogenesis and steroidogenesis alterations after chronic exposure to a human-relevant mixture of environmental toxicants spare the ovarian reserve in the rabbit model
Source: J Ovarian Res. 2024 Jun 28;17:134. doi: 10.1186/s13048-024-01457-6 (PMC11214233; doi:10.1186/s13048-024-01457-6)
Supplement: Supplementary file 1 — Supplementary Material 1 [file 13048_2024_1457_MOESM1_ESM.docx]

| **Accuracy** (%) | **Analytes** | **Target (GC-MS) or Precursor ion analyte (GC-MS/MS) / IS**  (m/z) | **Range**  (pg/ml) | **Mean** (25 runs; pg/ml)  ***Intra- & Inter assay CVs*** *(%)* | | | |
| --- | --- | --- | --- | --- | --- | --- | --- |
|  |  |  |  | **LLOQ**  Mean  Intra- & Inter assay CVs | **Low QC**  Mean  Intra- & Inter assay CVs | **Middle QC**  Mean  Intra- & Inter assay CVs | **High QC**  Mean  Intra- & Inter assay CVs |
| 95 ˗ 109 | T | 482 / 485 | 3.9 ˗ 944 | 3.6 | 153.7 | 310.4 | 621.5 |
|  |  |  |  | *15.1 ˗ 18.8* | *3.7 ˗ 5.6* | *3.6 ˗ 4.9* | *3.4 ˗ 5.1* |
| 94 ˗ 107 | E2 | 660 / 664 | 0.2 ˗ 56.0 | 0.22 | 2.97 | 6.11 | 12.08 |
|  |  |  |  | *17.3 ˗ 19.8* | *4.3 ˗ 5.7* | *3.6 ˗ 5.2* | *3.3 ˗ 5.4* |
| 95 ˗ 110 | E1 | 464 / 466 | 1.4 ˗ 111.0 | 1.4 | 25.2 | 48.9 | 100.9 |
|  |  |  |  | *18.1 ˗ 19.4* | *6.4 ˗ 8.7* | *5.3 ˗ 5.9* | *4.8 ˗ 5.8* |
| 95 ˗ 108 | DHEA | 482 / 485 | 16.5 ˗ 4000 | 17.6 | 297.5 | 595.8 | 1197.1 |
|  |  |  |  | *13.4 - 18.1* | *4.5 - 4.7* | *3.6 - 3.8* | *3.4 - 3.7* |
| 93 ˗ 110 | 4-dione | 482 / 488 | 11.7 ˗ 2833 | 11.6 | 58.8 | 99.1 | 198.5 |
|  |  |  |  | *16.3 ˗ 19.2* | *4.4 ˗ 5.2* | *3.5 ˗ 5.3* | *3.5 ˗ 5.9* |
| 94 ˗ 105 | Prog | 510 / 518 | 16.5 ˗ 4000 | 16.7 | 63.4 | 125.8 | 251.1 |
|  |  |  |  | *15.9 ˗ 19.5* | *3.3* *˗ 5.3* | *3.2* *˗ 5.3* | *3.0* *˗ 5.5* |
| 89 ˗ 113 | Preg | 298 / 302 | 25 ˗ 6000 | 25.8 | 62.6 | 127.9 | 248.9 |
|  |  |  |  | *16.1 ˗ 19.4* | *4.3 ˗ 6.7* | *4.1 ˗ 5.3* | *4.6 ˗ 5.8* |

**Supplemental Table S1.** **GC-MS analytical control validation.**

*LLOQ : low limit of quantification; QC : quality control*
